# Widespread distribution and a new recombinant species of Brazilian virus associated with cotton blue disease

**DOI:** 10.1186/1743-422X-5-123

**Published:** 2008-10-20

**Authors:** TF Silva, RL Corrêa, Y Castilho, P Silvie, J-L Bélot, MFS Vaslin

**Affiliations:** 1 Laboratório de Virologia Molecular Vegetal, Depto. Virologia, IMPPG, Universidade Federal do Rio de Janeiro, Rio de Janeiro, Brazil; 2 Departamento de Genética, I. Biologia, UFRJ, Rio de Janeiro, Brazil; 3 Centre de Coopération Internationale en Recherche Agronomique pour le Développement, CIRAD, Brazil, Brasilia, DF, Brazil; 4 Current Address: IRD, Institut de Recherche pour le Développement, CIRAD, Centre de Coopération Internationale en Recherche Agronomique pour le Développement, UR Systèmes de culture annuels (URSCA), Montpellier, F 34398, France

## Abstract

**Background:**

Cotton blue disease (CBD), an important global cotton crop pathology responsible for major economic losses, is prevalent in the major cotton-producing states of Brazil. Typical CBD symptoms include stunting due to internodal shortening, leaf rolling, intense green foliage, and yellowing veins. Atypical CBD symptoms, including reddish and withered leaves, were also observed in Brazilian cotton fields in 2007. Recently, a *Polerovirus *named Cotton leafroll dwarf virus (CLRDV) was shown to be associated with CBD.

**Results:**

To understand the distribution and genetic diversity of CLRDV in Brazil, we analyzed 23 CBD-symptomatic plants from susceptible cotton varieties originating from five of the six most important cotton-growing states, from 2004–2007. Here, we report on CLRDV diversity in plants with typical or atypical CBD symptoms by comparing viral coat protein, RNA polymerase (RdRp), and intergenic region genomic sequences.

**Conclusion:**

The virus had a widespread distribution with a low genetic diversity; however, three divergent isolates were associated with atypical CBD symptoms. These divergent isolates had a CLRDV-related coat protein but a distinct RdRp sequence, and probably arose from recombination events. Based on the taxonomic rules for the family *Luteoviridae*, we propose that these three isolates represent isolates of a new species in the genus *Polerovirus*.

## Background

Cotton (*Gossypium spp.*) is one of the most economically important crops in the world. Among its biotic pathologies, cotton blue disease (CBD) plays an important role due to its worldwide distribution and the high-magnitude of its associated productivity losses. Cotton blue disease was first described in the Central African Republic in 1949. It is transmitted by the aphid *Aphis gossypii *and its symptoms include leaf rolling, intense green foliage, vein yellowing, and a severe to moderate stunting caused by internodal shortening. Similar symptoms have also been observed in cotton crops of several regions of Africa, Asia and the Americas [1]. In Brazil, CBD is a serious crop problem capable of reducing the productivity of susceptible varieties by up to 80%, if intensive insecticidal control is not properly performed during the growing season. Losses of up to 1,500 kg·ha^-1 ^of cotton seed have been reported in some states [2].

The viral origin of CBD was only recently identified [3]. In Brazil, the disease is associated with a virus from the family *Luteoviridae*, genus *Polerovirus*, named Cotton leafroll dwarf virus (CLRDV) [3]. Viruses from the family *Luteoviridae *are icosahedral and have a positive-sense RNA genome of ~6 Kb. These viruses are transmitted by aphids in a circulative and persistent manner [4], and are restricted to the phloem cells of their hosts. The *Luteoviridae *family is comprised of three genera, *Luteovirus*, *Polerovirus*, and *Enamovirus*, that are divided based on differences in their RNA-dependent RNA-polymerase (RdRp) and structural proteins.

*Polerovirus *genomes consist of six open reading frames (ORFs), typically designated ORFs 0 to 6 [5]. Non-structural genes are located in the 5' portion of the genome. ORF0 encodes a silencing suppressor protein, P0 [6], and ORFs1 and 2 encode proteins related to viral replication, including the viral RNA polymerase [5]. Between the non-structural and structural protein sequences exists an intergenic region (IR) involved in subgenomic RNA synthesis [5,7,8]. The major coat protein (CP) is encoded by ORF3 at the 3' portion of the genome. The viral movement protein is encoded by ORF4, which lies within the CP gene sequence but in another reading frame [9]. ORF5 is expressed by occasional suppression of the CP termination codon [10] and encodes the read-through domain (RTD) required for efficient aphid transmission [11,12]

The genetic variability of plant viral populations is an important aspect of their evolution and epidemiology [13]. Variation among isolates may affect virulence, infectivity, transmission, and symptom severity, and therefore is important to consider when developing strategies for disease control [14]. To better understand the distribution and genetic diversity of CLRDV in Brazil, we analyzed CBD-symptomatic plants from susceptible cotton varieties obtained from five of the six most important cotton-growing states of the country. This work represents the first time that isolates of CBD-associated viruses have been analyzed at the molecular level.

## Materials and methods

### Plant material

**Figure 1:**
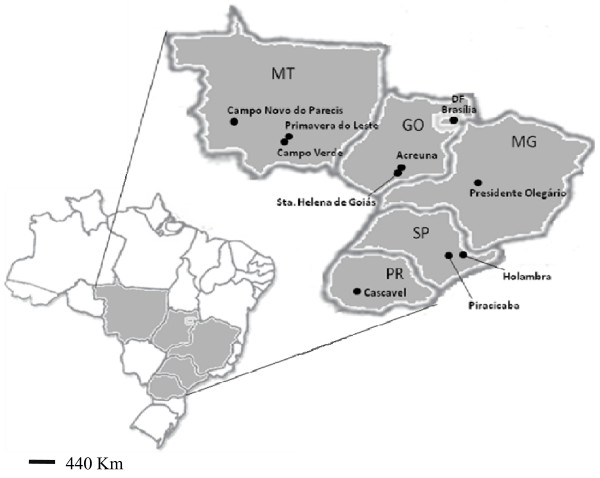
**Geographical map of Brazil indicating the districts where cotton samples were harvested.** DF, Federal District; GO, Goiás; MT, Mato Grosso; MG, Minas Gerais; PR, Paraná; and SP, São Paulo.

Twenty one cotton plants belonging to five susceptible cultivars of *Gossypium hirsutum *and two plants of *G. barbadense *were collected from cotton fields in the states of Mato Grosso, Goiás, Minas Gerais, São Paulo, Paraná, and Federal District from 2004 to 2007 (Table 1 and Figure 1).

**Table 1:** Locations, symptoms, and cotton species and cultivars of the 23 CLRDV isolate samples analyzed.

**Location**^1^	**Cotton sp.**	**Cultivar**	**Isolate**	**Date**	**Symptom**^2^	**Nested PCR**
Cascavel – PR	*G. hirsutum L.*	CD401xFM966	Cas1*	2004	typical	+
Cascavel – PR	*G. hirsutum L.*	FM966	Cas2*	2004	typical	+
Cascavel – PR	*G. hirsutum L.*	CD406XFM966	Cas3*	2004	typical	+
Cascavel – PR	*G. hirsutum L.*	CD406xFM966	Cas4*	2004	typical	+
Cascavel – PR	*G. hirsutum L.*	CD406xFM966	Cas5*	2004	typical	+
Sta. Helena de Goiás – GO	*G. hirsutum L.*	CNPA ITA 90	STG	2005	typical	+
Piracicaba – SP	*G. hirsutum L.*	CNPA ITA 90	Pir1	2005	typical	+
Piracicaba – SP	*G. hirsutum L.*	CNPA ITA 90	Pir2	2005	typical	+
Piracicaba – SP	*G. hirsutum L.*	CNPA ITA 90	Pir3	2005	typical	+
Piracicaba – SP	*G. hirsutum L.*	CNPA ITA 90	Pir4	2005	typical	+
Piracicaba – SP	*G. hirsutum L.*	CNPA ITA 90	Pir5	2005	typical	+
Piracicaba – SP	*G. hirsutum L.*	CNPA ITA 90	Pir6	2005	typical	+
Brasília – DF	*G. barbadense L.*	na	DF1	2005	typical	+
Brasília – DF	*G. barbadense L.*	na	DF2	2005	typical	+
Acreuna – GO	*G. hirsutum L.*	ST 474	Acr10	2006	typical	+
Presidente Olegário -MG	*G. hirsutum L.*	ST474	PO1	2007	atypical	+
Holambra – SP	*G. hirsutum L.*	nd	Hol1	2007	typical	+
Primavera do Leste – MT	*G. hirsutum L.*	FM966	PL2	2007	typical	+
Primavera do Leste – MT	*G. hirsutum L.*	FM966	PL3	2007	atypical	+
Primavera do Leste – MT	*G. hirsutum L.*	FM966	PL4	2007	typical	+
Campo Verde – MT	*G. hirsutum L.*	FM966	CV1	2007	typical	+
Campo Verde – MT	*G. hirsutum L.*	FM966	CV2	2007	atypical	+
Campo Novo do Parecis – MT	*G. hirsutum L.*	FM977	CNP1	2007	typical	+

* Samples inoculated in the Coodetec Research Center greenhouse. na, not applied; nd, not determined. ^1^DF, Federal District; GO, Goiás state; MT, Mato Grosso state; MG, Minas Gerais state; PR, Paraná state; and SP, São Paulo state. ^2^Symptoms description: typical, internodal shortening, leaf rolling, intensive green foliage and vein yellowing; atypical, typical disease symptoms plus withered and reddish basal leaves.

### RNA extraction and amplification

The total RNA was extracted from leaf samples using the RNeasy Plant Mini kit (Qiagen) in combination with a borate extraction buffer, following a previously described procedure [15]. Approximately 2.5 μg of total RNA from each isolate were used to synthesize first-strand cDNA using the CLRDV-specific primer O5R2 (5'-GCAACCTTTTATAGTCTCTCCAAT-3'), which anneals in the middle of CLRDV ORF5. Two independent nested PCRs were carried out for each viral isolate, one to amplify the CP gene and the other to amplify part of the RdRp gene plus the complete IR.

To amplify the CP gene, an initial reaction with the primers PL2F [3] and O5R2 was performed. The obtained amplicon was used as a template for a second amplification with the nested primers CPF and CPR [3], generating a 650-nt fragment. To amplify the partial RdRp sequence plus the IR, the previously described degenerated primers PLF and PLR [3] were used in the first reaction. Following this, the internal primers PL2F and PL2R [3] were used in the second reaction, generating a 468-nt fragment. Reactions were carried out with a denaturation step of 5 min at 95°C followed by 40 cycles (for the pairs PLF, PLR and PL2F, PL2R) or 35 cycles (for the primers PL2F, O5R2 and CPF, CPR) at 95°C for 1 min, 52°C (for PL2F, O5R2), 65°C (for CPF, CPR and PLF, PLR), or 50°C (for PL2F, PL2R) for 1 min, and 72°C for 1 min, and a final extension step at 72°C for 10 min. To amplify the full-length fragment corresponding to the partial RdRp, IR, and CP regions in a single PCR product, PL2F and CPR were used with an annealing temperature of 52°C for 40 cycles. RT-PCR products were purified using the Wizard^® ^SV Gel and PCR Clean-UP System (Promega) following the instructions provided.

### Sequencing and sequence analysis

Three amplicons of each viral fragment, derived from independent RT-PCR reactions, were purified and then directly sequenced in both directions in automated ABI sequencers (models 310 or 377) using dye terminator cycle sequencing for all isolates. The resulting sequences were compared with the GenBank database. Consensus sequences of the CP, partial RdRp, and IR were obtained through the Multalin program [16].

Multiple sequence alignments of nucleotide or deduced amino acid sequences were submitted to the GeneDoc program  for computational analysis of the identities between the sequences. Phylogenetic reconstructions were performed using the MEGA 4 software [17]. Trees were constructed by the neighbor-joining (NJ) method [18]. The pair-wise deletion option, excluding gaps from the sequence alignment, and p-distance matrix were adopted. Data sets were bootstrapped (1,000 replicates) to assess the confidence values of the phylogenetic trees, and bootstrap values < 50% were omitted. *Turnip yellows virus *(TYV), which represents the species most closely related to CLRDV across the polymerase and coat protein sequences, was included as an out-group.

The following sequences from members of the family *Luteoviridae *were used in the phylogenetic analyses: *Carrot red leaf virus *(CRLV) AY695933, *Cereal yellow dwarf virus-RPV *(CYDV-RPV), L25299; *Cereal yellow dwarf virus-RPS *(CYDVRPS), AF235168; *Chickpea stunt disease associated virus *(CpSDaV), AY956384; TYV, X13063; *Beet mild yellowing virus *(BMYV), X83110; *Beet chlorosis virus *(BChV), AF352024; *Beet western yellows virus *(BWYV), AF473561; *Cucurbit aphid-borne yellows virus *(CAbYV), X76931; *Potato leafroll virus *(PLRV), D00530; *Soybean dwarf virus *(SbDV), AB038147; *Tobacco vein distorting virus *(TVDV), AF402621; *Bean leafroll virus *(BLRV), AF441393; *Barley yellow dwarf virus-PAV *(BYDV-PAV), X07653; *Barley yellow dwarf virus-PAS *(BYDV-PAS), AF218798; *Barley yellow dwarf virus-GAV *(BYDV-GAV), AY220739; *Barley yellow dwarf virus-MAV *(BYDV-MAV), D01213; *Sugarcane yellow leaf virus *(ScYLV), AF157029; and *Pea enation mosaic virus-*1 (PEMV-1), L04573.

The occurrence of recombination events was investigated by the programs Simplot [19], Genetic Algorithms for Recombination Detection (GARD) [20], and bootscans implemented by Recombination Detection Program version 2.0 (RDP2) [21]. To further analyze recombination events, sequences obtained from individual PCRs were concatenated *in silico *and used in the above-described software. For Simplot analysis, the two previously described sequences of CLRDV (AY758560 and AY758561) were used as queries against the reference sequences of the isolates PL3, CV2, and PO1 and a cluster of sequences composed of all other sampled CLRDV isolates. The identities were calculated in a sliding window of 120 bp, which was moved across the alignment in 10 bp steps. The parameters used in the GARD program for checking alignments of CLRDV and the divergent isolates were the multiple break points options and general discrete rate variation. A manual bootscan was implemented by RDP3 Beta 24, with window and step size parameters of 100 and 10 bp, respectively. A 70% bootstrap support was considered to be definitive.

## Results

To analyze the ubiquity and diversity of CBD in Brazil, cotton plants showing typical symptoms were harvested from commercial and experimental fields in four of the six major cotton-producing states of Brazil, Mato Grosso (MT), Goiás (GO), São Paulo (SP), Paraná (PR), and at Federal District (DF), between 2004 and 2007 (Figure 1). Three plants with atypical CBD symptoms were also harvested in the states of Minas Gerais (MG) and MT. Plants with atypical symptoms had all of the typical CBD symptoms along with withered and reddish middle and basal leaves.

Using nested RT-PCR, we tested for the presence of CLRDV-related sequences in all 23 sampled plants. The CP sequence, part of RdRp, and the full IR were amplified in all the isolates tested (Table 1). All amplicons showed the expected size for CLRDV and were sequenced. At least three independent PCR reactions for each fragment from each sample were sequenced. The obtained sequences were aligned and a consensus nucleotide sequence for each isolate was made for the three viral fragments.

The CP sequences of all isolates were similar to the CP sequence from CLRDV deposited in the GenBank database (accession number AY758560). In addition, most of the RdRp sequences analyzed were also closely related to that of the previously reported isolate (accession number AY758561). However, three isolates (designated PL3, CV2, and PO1) showed best hit results with TVDV, an unclassified member of the family *Luteoviridae*, but not with CLRDV. This suggests that these three isolates may have resulted from recombination events. Interestingly, these three divergent viruses were found in plants with atypical CBD symptoms.

When we compared CP sequence identities from all the isolates, we observed a high degree of nucleotide identity (97–100%). However, when the partial RdRp sequences were analyzed, the sequence identities were lower (65–100%). Most of this diversity resulted from a high sequence divergence in the three isolates found in plants with atypical CBD symptoms. Isolates PL3, CV2, and PO1 shared identities of 68%, 68%, and 67%, respectively, with the original CLRDV RdRp sequence. The identities among PL3, CV2, and PO1, however, ranged from 93% to 95%. Excluding the divergent isolates from the analysis, identities ranging from 95 to 100% for the RdRp sequence were observed among the 20 newly-identified CLRDV isolates. Similar results were also obtained for the IR, where the identities ranged from 94% to 100% among the 20 CLRDV isolates. The three divergent isolates shared identities of 66% with the original CLRDV IR sequence.

### Phylogenetic relationships among isolates

**Figure 2:**
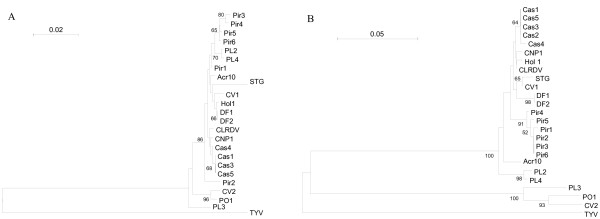
**Phylogeny of the viral isolates.** (A) Phylogenetic trees were constructed based on nucleotide sequence alignments of the CLRDV CP coding region, 606 nt, or (B) on the partial RdRp coding region, 280 nt. Numbers above the lines indicate the bootstrap scores out of 1,000 replicates. The tree was constructed with MEGA4 and the scale bar represents genetic distance. The TYV sequence used was deposited in the GenBank database under the accession number X13063.

Phylogenetic relationships among the CLRDV isolates were determined using the NJ method. Since dendograms constructed from nucleotide or amino acid sequences produced similar results, only those derived from nucleotide sequences are shown (Figure 2). In general, CLRDV displayed a widespread distribution in Brazil. Two groups of CP phylogeny were found: a small cluster formed by isolates CV2 and PO1 (bootstrap of 96%) and a large cluster containing almost all of the other sampled isolates (bootstrap of 86%). PL3 appeared to be the most divergent isolate, since its CP sequence was not grouped with the others. Within the major cluster were clusters of viruses from the same locality. This was particularly observed in plants harvested in Cascavel, PR and in DF during the 2004 and 2005 harvests, and in those obtained from Primavera do Leste, MT in 2007 (Figure 2A).

The trees constructed from partial RdRp (Figure 2B) and IR (data not shown) sequences were congruent and revealed a clear segregation of the isolates into two monophyletic clusters. This dichotomy was supported by 100% bootstraps in trees constructed either on nucleotide- or amino acid-based alignments (data not shown). The first cluster contained the three divergent isolates, PL3, CV2, and PO1, while the second was comprised of all the remaining isolates. Again, there were clusters of isolates obtained from the same geographical region in the same harvest. The isolates from Cascavel that were harvested in 2005 grouped together, as did those from DF (2005) and Primavera do Leste (2007) (Figure 2B).

The three divergent isolates were clearly separated from the other 20 CLDRV isolates found. Although these three isolates were very close to the other isolates, the CP phylogenetic tree placed them in an independent cluster. Significant differences were found between RdRp sequences from the divergent isolates and those from viruses associated with typical CBD symptoms, suggesting the possibility of recombination events between the viral populations in the field.

### Characterization of the divergent isolates

**Figure 3:**
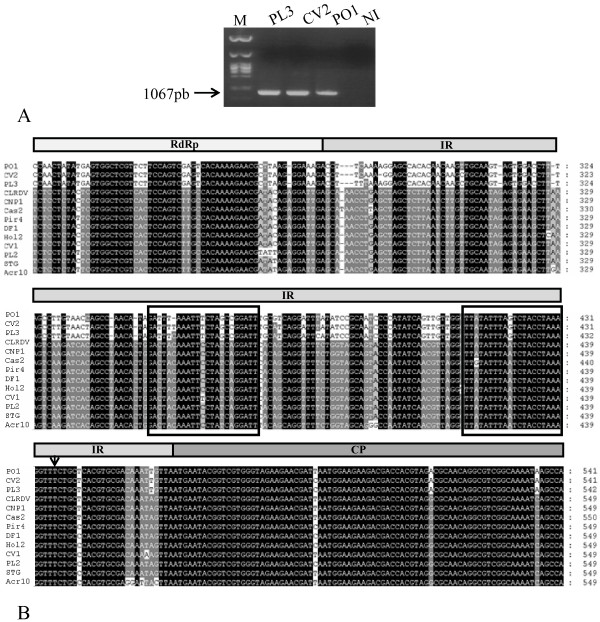
**Amplification and alignment of the divergent nucleotide sequences. **(A) Amplification of a single amplicon (1074 bp) corresponding to the partial RdRp, IR, and CP regions of isolates PO1, PL3, and CV2. NI, non-infected plant; M, λ-DNA digested with *Pst*I. (B) Alignment of isolates PO1, PL3, and CV2 with a CLRDV isolate from each of the Brazilian regions analyzed in this work. Black boxes represent residues present in all viruses in the alignment. Columns in the alignment with < 100% but > 60% conservation are shaded in gray. The arrow represents the break point indicated in the GARD analysis (see Figure 6). Sequences inside the black boxes are associated with subgenomic RNA formation [7].

To characterize the divergent isolates, we first checked whether the sequences of two different viruses were misamplified in a co-infection context. Using a primer combination capable of amplifying a fragment comprised of the 3' end of RdRp plus the IR and CP sequences, we obtained a single PCR product with the expected size for each divergent isolate (PL3, CV2, and PO1) (Figure 3A). The amplified fragments were cloned and sequenced, and the new sequences confirmed that each isolate corresponded to the amplification of a single viral genome.

The sequences obtained were aligned with the sequences of CLRDV and nine other randomly chosen isolates. All the viruses had almost identical CP sequences. Similarities were also observed in the 3' end of the IR. However, the 5' end of the IR and the 3' portion of the RdRp sequence showed consistent differences (Figure 3B).

**Figure 4:**
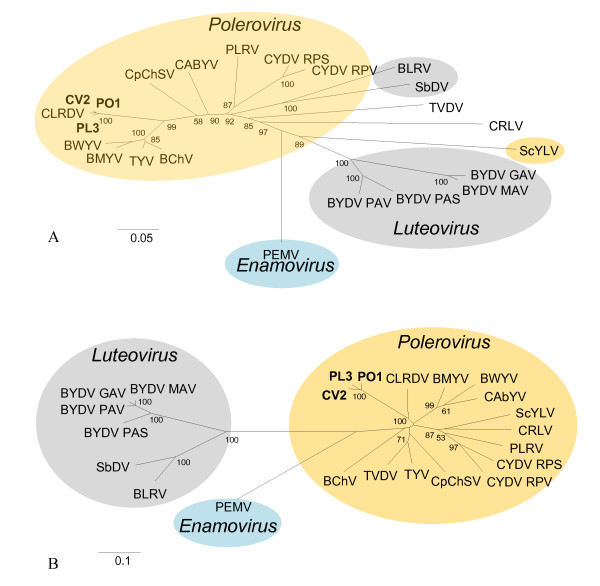
**Phylogenetic analysis of isolates PO1, PL3, and CV2 and other viruses from the family *Luteoviridae*.** (A) The predicted amino acid sequences of the CP and (B) the C-terminal region of RdRp were aligned with Multalign and the trees were constructed with MEGA4. Bootstrap values are indicated at each node of the trees. GeneBank accession numbers for the sequences are listed in the Materials and Methods section. *Luteovirus*, *Polerovirus*, and *Enamovirus *members are distinguished with grey, orange, and blue circles, respectively. *Carrot red leaf virus *(CRLV) and *Tobacco vein distorting virus *(TVDV) are unclassificated *Luteoviridae *members with *Polerovirus*-like RdRps.

To better understand the taxonomic and evolutionary positions of isolates PL3, CV2, and PO1 within the family *Luteoviridae*, sequences of the three viruses were compared to well-characterized *Luteoviridae *members. When the deduced amino acid sequence of the CP gene was analyzed, identities of 97%, 79%, 78%, and 77% were observed with CLRDV, TYV, BMYV, BChV, and BWYV, respectively. Phylogenetic analysis grouped the three divergent isolates with CLRDV in the *Polerovirus *branch of the tree, together with other definitive members of this group (Figure 4A).

The partial RdRp sequence analyzed in this work encodes the 92 C-terminal residues of the viral protein. Although the RdRp sequences of the three recombinant isolates shared an almost 70% sequence identity with CLRDV, significant identities were also found with *Polerovirus *members, including TYV (68%) and BMYV (66%), and with TVDV (66%), an unclassified *Luteoviridae *that has a *Polerovirus*-like RdRp. Identities with other known *Luteoviridae *viruses indicated that PL3, CV2, and PO1 are more related to members of the genus *Polerovirus *than to those of *Luteovirus *(data not shown). Phylogeny obtained from the partial RdRp amino acid alignment confirmed this result by grouping the three isolates with CLRDV in the *Polerovirus *branch (Figure 4B).

Together, the above results indicate that PL3, CV2, and PO1 should be regarded as definitive members of the family *Luteoviridae*, genus *Polerovirus*. Considering the high degree of sequence divergence in the RdRp region, these three isolates may also represent isolates of a new species in *Luteoviridae *and of a second CBD-associated virus in Brazil.

### Recombination analysis

**Figure 5:**
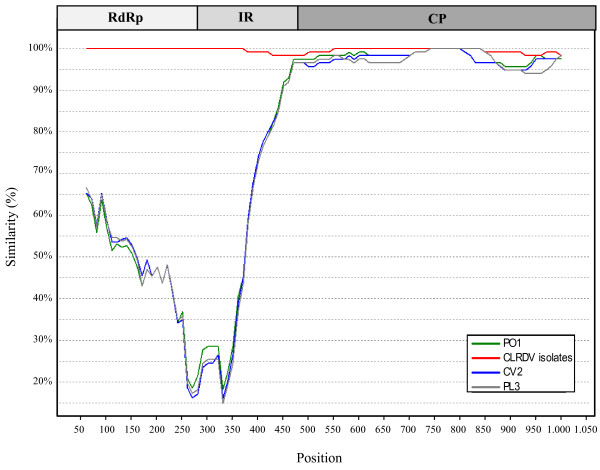
**Simplot analysis of the three divergent isolates (PO1, PL3, and CV2) and CLRDV isolates.** Full-length sequences, comprising the partial RdRp, IR, and CP regions, were analyzed. The horizontal axis represents the nucleotide distance of the midpoint of the window from the 5' end of the query sequence (1074 nt in CLRDV). The vertical axis represents the percentage of similarity (within a window covering 120 bp). The window was moved through the alignment with a step length of 10 bp.

To identify recombination events that could explain the emergence of the PL3, CV2, and PO1 isolates, the RdRp, IR, and CP sequences from each of the 23 isolates were analyzed using Simplot. For the 20 CLRDV isolates, the analyses were carried out by *in silico *concatenation of the individual sequences obtained by PCR. Similar to the above results, a comparison of the RdRp sequences revealed that these three isolates had low sequence similarities to CLRDV and to the other 20 isolates (Figure 5). However, a pronounced increase in the similarities between PL3, CV2, and PO1 and the other CLRDV isolates was observed from the middle of the IR to the end of the CP sequence (Figure 5).

**Figure 6:**
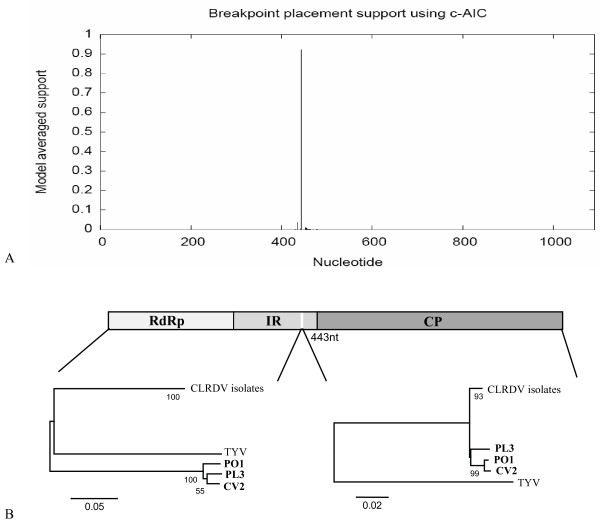
**Breaking point detection in PO1, PL3, CV2, CLRDV isolates and TYV alignment.** (A) Detection of a breaking point at nt 443 of the alignment by GARD, using AIC as a criterion. The vertical axis displays the model-averaged probability of finding a break point at a given position in the alignment, represented in the horizontal axis. (B) Partial genome representation, indicating the break point and the phylogenies proposed by GARD for the segments before and after nt 443. The phylogenetic relationship between all other CLRDV isolates was collapsed and represented as a single branch designated "CLRDV isolates". Scale bar represents genetic distance. The TYV sequence used was deposited in the database under the accession number X13063.

The hypothesis that the divergent isolates resulted from recombination events was reinforced by results obtained using GARD. A discordant phylogenetic signal in the alignment of PL3, CV2, PO1, the 20 other concatenated CLRDV isolates, and TYV nucleotide sequences indicated a clear breaking point at nucleotide (nt) 443 (Figure 6A). The proposed breakpoint-delimited phylogenetic tree revealed that the cluster formed by PL3, CV2, and PO1 was more divergent to CLRDV isolates than to those of the TYV out-group, when the 5' portion of the alignment was considered (Figure 6B). However, for a segment downstream of the breakpoint, the phylogeny showed a grouping of the clusters formed by the divergent and CLRDV isolates.

**Figure 7:**
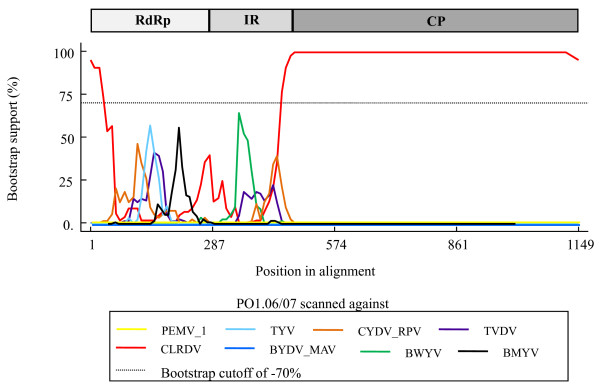
**Bootscan analysis of the full-length sequence of the isolate PO1 against *Luteoviridae *members.** The horizontal axis represents the nucleotide distance of the midpoint of the window (covering 100 pb). The vertical axis represents the percentage of trees (using 100 bootstrap replicates) that support branching with the divergent isolate query sequence. The values for one *Enamovirus *(PEMV-1), one *Luteovirus *(BYDV-MAV), the four *Polerovirus *with the highest bootstraps (TYV, CYDV-RPV, BWYV, and BMYV), and TVDV (an unclassified member of the family) are represented. The GenBank accession numbers for these sequences are listed in the Material and Methods section.

Nucleotide 443 is located within the IR, a well-known site of both intra- and inter-species recombination in the family *Luteoviridae*. To identify possible parental lines for the divergent isolates, a bootscan analysis was performed. *Luteoviridae *sequences corresponding to the partial RdRp, IR, and capsid gene were used to scan for homologue segments in PL3, CV2, and PO1. For the RdRp and IR sequences of PO1, the bootstrap values were low until ~nt 435 and did not support their clustering with CLRDV (Figure 7). However, no significant bootstrap values were reached to support the clustering of the divergent sequences with any other luteovirus. For CP and nearby sequences, the bootstrap values supported the grouping of the divergent isolates with CLRDV (Figure 7), as expected. Similar plots were obtained for isolates CV2 and PL3 (data not shown).

## Discussion

Analyzing CLRDV distribution in cotton Brazilian fields we were able to shown that it has a widespread distribution and high sequence conservation. These conclusions arise from nucleotide sequence comparing part of the polymerase, the coat protein and the intergenic region of 23 new isolates and CLRDV isolate identified in 2005 in Primavera do Leste. Interestingly, we also found that three of them have enough divergence in their polymerase sequences to be considered as a new species.

Cotton blue disease is widely distributed in Brazil and throughout the world; however, due to the recent characterization of its causal agent at the molecular level, its viral population and/or isolate diversity have not previously been reported. We previously described a virus associated with CBD in Brazil [3]. This virus, CLRDV, was present in CBD-symptomatic plants from a crop field in Primavera do Leste, MT. Here we map for the first time the genetic diversity of CLRDV in Brazilian cotton fields covering the most important producer regions. Analyzing 23 new viral isolates, we observed a low diversity in the CP CLRDV gene. However, phylogenetic analysis of this sequence revealed a small genetic distance between CV2, PO1, and PL3 and the other CLRDV isolates, clustering them in different groups. These results may suggest a co-adaptation phenomenon, resulting from selection pressure, among the divergent and the other CLRDV isolates, giving rise very similar CP sequences. Indeed, evolutionary studies concerning the CP of *Luteoviridae *members show that this protein is subjected to various selection pressures [22]. The CP is directly associated with the success of infection, as it is involved in viral transmission, particle packaging, and viral accumulation within the plant [23,24]. Thus, a high degree of conservation in the CP protein sequence is expected.

*Luteoviridae *members have varying degrees of sequence conservation along their RNA genome. A model has been proposed where the CP and RTD sequences are the most conserved, while the IR is the least. Accordingly, our results showed that the partial RdRp CLRDV gene displayed a greater diversity than CLRDV CP. Our analysis of the IR, however, contradicted this model, and showed a low diversity in this region comparable to that of the RdRp sequence. The IR is a noncoding sequence, and should be more prone to sequence variation than other parts of the genome. However, its role as a promoter in the generation of the subgenomic RNAs could result in sequence conservation due to structural constraints [7,8].

Interestingly, the isolates amplified from plants displaying typical CBD symptoms were very similar with one another, but those associated with atypical symptoms (PL3, CV2, and PO1) displayed highly divergent RdRp sequences compared to the previously published CLRDV sequence. A phylogenetic analysis of the partial RdRp region revealed a clear segregation between PL3, CV2, and PO1 and the other CLRDV isolates identified in this study, forming two monophyletic clusters. This segregation was partially observed in the phylogeny of the CP gene, although the dendograms for this sequence revealed a stronger relationship among the divergents and the other CLRDV isolates.

Taxonomic characterization of the divergent isolates using phylogenetic analyses indicated that PL3, CV2, and PO1 have a *Polerovirus*-like RdRp and a CLRDV-like CP. Moreover, the IR between the RdRp and CP sequences from divergent isolates have 187 nt, the same as that in CLRDV [3]. Thus, their IRs resemble those of *Polerovirus *members, which are ~200 nt long, and differ from those of *Luteovirus *members, which are ~100 nt long. Taken together, these data suggest that the three divergent isolates could result from recombination events between CLRDV ancestors and another member of the genus *Polerovirus*. Evidence of recombination was found using three different methods (Simplot, GARD, and Bootscan). However, we were unable to detect a parental sequence for PL3, CV2, or PO1. Thus, the *Polerovirus *member involved in recombination with CLRDV remains unknown.

Recombination events may play an important role in generating genome diversity. Inter-species recombination has frequently occurred in the evolution of *Luteoviridae*. RNA recombination event(s) probably created the divergence observed between the genera *Luteovirus *and *Polerovirus *[25]. Subsequent recombination events between polerovirus-polerovirus [26] and luteovirus-polerovirus [27,28] have occurred since the birth of these genera [5,25]. Recombination sites in the family appear to coincide with the starting points for subgenomic RNA synthesis in the IR [7]. Comparisons among the IR sequences of BWYV, PLRV, and BYDV RNAs revealed two spots that are apparently conserved in all viruses. These regions contain a repeated U_n_A signature that is usually associated with the formation of subgenomic RNAs [7,8]. Our data suggest that the recombination event found in the three divergent isolates occurred downstream of the second recombination spot of the IR (Figure 3B). Consistent with this, crossover events at the same region have already been described for a bovine coronavirus [29].

Amino acid sequence comparisons between the three recombinant viruses isolated from plants with atypical CBD symptoms and the 20 analyzed CLRDV isolates revealed that they share identities of ~70% in the partial RdRp sequence and of ~97% in the CP region. The molecular criterion used for species discrimination [30] in the family *Luteoviridae *states that amino acid sequences from any viral gene product exceeding 10% divergence should be regarded as a new species. Furthermore, symptomatology differences are also an important criterion for species discrimination. Therefore, our results suggest that PL3, CV2, and PO1 should be considered isolates of a new species in the genus *Polerovirus*. Further studies on CBD are necessary to understand the taxonomic and epidemiological relationships between the viruses associated with this important disease.

## Conclusion

Our data suggests a CLRDV low genetic diversity and widespread distribution in Brazilian states. Between the 23 samples analyzed, however, we identified three divergent isolates associated with atypical CBD symptoms. The analyses revealed that these isolates have a CLRDV-related coat protein but distinct RdRp sequences and probably arose from recombination events between CLRDV and an unidentified luteovirus. In agreement with molecular taxonomic criterions for the *Luteoviridae*, we can consider that these isolates represent a new specie in the genus *Polerovirus*.

## Competing interests

The authors declare that they have no competing interests.

## Authors' contributions

TFS carried out the molecular genetic studies, participated in the sequence alignment, the recombination analysis and drafted the manuscript. RLC participated in the design of the study, in the design of primers, in the sequence alignment. YC performed RT-PCR and NESTED assays. Sample identification and collection in field were carried out by PS and J-LB. They also participate in the design of the study. MFSV conceived of the study, and participated in its design and coordination and helped to draft the manuscript. All authors read and approved the final manuscript.
